# Maternal and Infant Skin Microbiome Synchrony and Divergence: A Longitudinal Study of Skin Microbial Ecology and Early‐Life Assembly

**DOI:** 10.1111/exd.70349

**Published:** 2026-08-21

**Authors:** Doris Wilborn, Andria Constantinou, Agathe Franz, Rolf Schwarzer, Peter Menzel, Gabriela Engelhardt, Tsenka Tomova‐Simitchieva, Ruhul Amin, Gavin Zhou, Kathrin Hillmann, Jan Kottner, Jann Ralle, Frank Konietschke, Ulrike Blume‐Peytavi

**Affiliations:** ^1^ Department of Dermatology, Venereology and Allergy Charité – Universitätsmedizin Berlin, Corporate Member of Freie Universität Berlin and Humboldt‐Universität zu Berlin Berlin Germany; ^2^ Labor Berlin Charité Vivantes GmbH Berlin Germany; ^3^ BCSIR Laboratories Dhaka Bangladesh Council of Scientific and Industrial Research Dhaka Bangladesh; ^4^ Dehaa Rossun Research Center of Lunaler Group Kowloon, Hong Kong China; ^5^ Institute for Clinical Nursing Science Charité – Universitätsmedizin Berlin, Corporate Member of Freie Universität Berlin and Humboldt‐Universität zu Berlin Berlin Germany; ^6^ Institute of Biometry and Clinical Epidemiology Charité – Universitätsmedizin Berlin, Corporate Member of Freie Universität Berlin and Humboldt‐Universität zu Berlin Berlin Germany

**Keywords:** environment, infants, pregnancy, skin characteristics, skin microbiome

## Abstract

During pregnancy and after childbirth, women's skin undergoes significant changes which may affect the skin's functional and structural characteristics and microbial diversity. Like their mothers, the newborns also experience various skin challenges in the months following birth. Temporal changes in skin microbiome in special groups such as pregnant women and newborns, as well as the interrelation between their skin's microbial diversity, have not been investigated. We followed women from pregnancy through 6 months after delivery, and their infants from 4 weeks to 6 months of age to investigate their skin characteristics and the microbiome, and potential associations. We enrolled 109 pregnant females residing in Berlin, Germany, with 93 mothers‐infant pairs completing the study. Microbiome investigations included DNA isolation as single‐site sampling using volar forearm skin swabs. Bioinformatic analysis involved OTU clustering at 97% sequence identity, taxonomic assignment using NCBI reference databases, and calculation of biodiversity metrics, including relative abundance of the dominant bacterial phylotypes, bacterial diversity and Shannon diversity index. The maternal skin microbiome showed mild to moderate changes throughout pregnancy and the 6 months postpartum period. In infants, alpha diversity significantly increased (mean species richness from 82.6 at 4 weeks to 116.1 at 6 months), although it did not reach maternal diversity levels (145.4 at 6 months postpartum). Regarding the microbiome consistency of the included women and infants, the within‐pair similarity was always significantly higher than between‐pair similarity for both time points. Therefore, we conclude that there is likely a relationship between the microbiome of the mother and that of her child.

**Abbreviations**
AD: Atopic dermatitis
DNA: Deoxyribonucleic acid
EEMCO: Expert group on efficacy measurement of cosmetics and other topical products
ICF: Informed consent form
ICH: International conference on harmonization of technical requirements for registration of pharmaceuticals for human use
IQR: Interquartile range
OCT: Optical coherence tomography
PCR: Polymerase chain reaction
pH: Pondus hydrogenii
RNA: Ribonucleic acid
SCH: Stratum corneum hydration
SD: Standard deviation
STROBE: Strengthening the reporting of observational studies in epidemiology
TEWL: Transepidermal water loss

## 1 Introduction

### 1.1 Background/Rationale

The human microbiome is defined as a community of commensal, symbiotic and pathogenic microorganisms within a body space or other environment [1]. In recent years, extensive investigations have described the cutaneous microbiome in healthy adults, but research on special groups such as pregnant women and infants is limited. Initial characterizations of the healthy adult skin microbiome revealed that the microbial composition varies by anatomical region [2]. Byrd et al. differentiated distinct major skin microenvironments as oily, moist and dry body regions and the foot [3]. On dry sites, like the volar forearm, the composition of the skin microbiome has the greatest diversity, with a mixed population of *Cutibacterium*, *Staphylococcus*, *Corynebacterium* and *Streptococcus* species [3]. At the phylum level, Grice et al. identified 18 total phyla, with the four dominants being *Actinobacteria* (51.8%), *Firmicutes* (24.4%), *Proteobacteria* (16.5%) and *Bacteroidetes* (6.3%) [2]. Another study shows that *Cutibacterium* predominantly colonizes dry sites in healthy adults [4].

While the adult skin microbiome shows a remarkable stability and individual specificity over years, comparable to a ‘fingerprint’ [4, 5], the infant skin microbiome undergoes great changes during development. Immediately after birth, bacterial colonization is uniform across multiple body skin areas [6, 7], with delivery mode greatly influencing initial microbial composition. More specifically, vaginally delivered newborns harbour bacterial communities like maternal vaginal microbiota, mainly *Lactobacillus*, *Prevotella* and *Atopobium*. In contrast, newborns delivered by caesarean‐section showed a higher abundance of typical skin taxa commensal like *Staphylococcus* spp. [6]. However, during the first 6 weeks of life, the infant skin microbiome was found to evolve primarily based on body site rather than by mode of delivery [7].

The infant skin was found to be predominantly colonized by *Firmicutes*, followed in abundance by *Actinobacteria*, *Proteobacteria* and *Bacteroidetes* [8], and in general, it was found to resemble that of adult moist skin regions [9]. So, the body region and age were found to be the primary factors determining the skin's bacterial composition, though when compared to the maternal cutaneous microbiome, notable similarities were found between mother–child pairs [10]. A study of children up to 10 years old and their mothers (aged 25–40) further demonstrated that the skin microbiome continues to mature during early childhood in a site‐specific manner [11]. *Firmicutes* was found to be the predominant phylum in the infant groups, while *Proteobacteria* was most predominant in adults. Another study, which focused on the dynamics of the skin microbiota between mothers and their infants during the first postpartum year, demonstrated temporal variations in microbial colonization patterns [12]. More specifically, they showed a peak in bacterial genera diversity occurred at 6 months postpartum for both mothers and infants. While certain taxa (*Proteus and S. aureus*) maintained stable isolation rates, others (*Enterobacter*, *Enterococcus*, *Klebsiella* and *Pseudomonas*) showed significant temporal variation. Notably, the progressively declining rates of the cultured genera throughout the first year were the same within the mother‐infant pairs. Thus, suggesting that during the first year postpartum, this might be a further hint that other factors beyond mother‐newborn transmission were responsible for the emerging skin microbial individuality of the growing infant [12]. In their study, Gaitanis et al. [12] used a culture‐based approach following 17 mother‐infant dyads up to 12 months postpartum. However, their study was limited to culturable bacteria and lacked compositional and diversity‐level resolution via sequencing approaches.

Many reports have addressed the potential of the skin microbiome in various activities and interactions with both the environment and the host itself [1], revealing that these activities and interactions are often dependent on specific body sites and their local conditions. For example, changes in the skin pH were associated with changes in the skin bacterial and fungal microbiomes at sebaceous sites [13], whereas positive correlations between the relative abundance (%) of *Corynebacterium* and *Staphylococcus aureus* and TEWL were shown in Korean healthy adults on the cheeks and forehead [14]. Other study results showed associations between the richness and diversity of the microbiome, the skin and environmental properties [6, 8, 15, 16]. This indicates complex relationships between physiological development, microbial colonization and growth and external influences.

Although in recent years, investigations have described the cutaneous microbiome in healthy adults and infants, special groups such as pregnant women and newborns have been largely overlooked, and possible associations between women and their infants remain underexplored. In particular, the dynamic longitudinal changes shaping these microbial communities during the perinatal period have yet to be characterized. While several studies have profiled adult skin microbiota or neonatal microbiota shortly after birth, few have addressed how maternal and infant skin microbiomes evolve in synchrony over a longitudinal period. Furthermore, no study has longitudinally tracked the skin microbiome from pregnancy through 6 months postpartum using high‐throughput sequencing.

Therefore, to further elucidate the dynamics of maternal–infant cutaneous bacterial microbiome development, we conducted an observational prospective cohort study using 16S rRNA sequencing to investigate the bacterial skin microbiome of pregnant women and their infants at week four and month six postpartum. We aimed to explore how maternal–infant microbiota relationships change over time and to identify potential associations with host or environmental factors.

### 1.2 Objectives

This study aimed to establish basic knowledge of the dynamics in infants' and women's bacterial skin microbiome to further understand its synchrony and divergence by characterizing the cutaneous bacterial microbiome of mothers and infants over time and identify individual, environmental and skin characteristics associated with its development.

The specific research questions were as follows:

1. What is the main bacterial composition of the maternal and infant skin microbiome during pregnancy and the postpartum period?
2. How does the maternal skin microbiome composition change from pregnancy through the postpartum period?
3. What temporal changes occur in infant skin microbiome between 4 weeks and 6 months postpartum?
4. What associations exist between maternal and infant skin microbiome in the first 6 months postpartum?
5. Which individual, environmental and cutaneous characteristics are associated with the maternal or infant skin microbiome respectively?

## 2 Methods

All participants gave their informed consent for inclusion before they participated in the study. The study was conducted in accordance with the Declaration of Helsinki, and the protocol was approved by the Ethics Committee of the Charité–Universitätsmedizin Berlin, Germany (Project identification code EA2/184/20).

### 2.1 Study Design

We performed a prospective observational cohort study including women during pregnancy, 4 weeks and 6 months postpartum and their infants from 4 weeks until 6 months of age to assess skin characteristics and the skin microbiome during that challenging period of life. Setting, participants, variables, data sources and measurements were briefly described in the study protocol [17]. Shortly, skin characteristics as pH value, stratum corneum hydration, TEWL, skin surface topography and epidermal thickness were collected during pregnancy, 4 weeks and 6 months after birth, as well as individual characteristics as age, week of pregnancy and environmental details. The definitions of all variables including time and units of measurement are shown in Table S1.

### 2.2 Sample Collection, Sequencing and Microbiome Analysis

The skin microbiome was characterized by analyzing the bacterial diversity and community composition through measuring the relative abundance of different bacterial groups at the phylum level, classified as operational taxonomic units (OTUs). Skin swabs were collected from the left volar forearm using a swab moistened with sterile NaCl 0.9% + 0.5% Tween 20 solution. After sampling for 30 s always avoiding any cross‐contamination, the swab head was added into a tube containing DNA/RNA Shield (Zymo Research, Irvine, USA) to preserve the nucleic acids at ambient temperature. All samples were stored at room temperature until laboratory analyses. We used the NCBI‐taxonomy of 2020 for defining the names of the identified phyla, order and genus in our microbiome sample [18]. All microbiome results of cited literature were presented in their original used taxonomy.

#### 2.2.1 Sequencing

We followed the analytic test methods from the Human microbiome project by McInnes and Cutting for describing the microbiome distribution of bacterial species in this study [19]. For the analysis, the V3‐V4 regions of the 16S rRNA gene were amplified and the amplicons were sequenced according to Illumina's 16S Metagenomic Sequencing Library Preparation Protocol with minor modifications [20]. For this purpose, DNA was isolated from the skin swab using the DNeasy PowerSoil Pro kit (Qiagen, Hilden, Germany) and eluted in 50 μL elution buffer. The 16S‐V3V4 PCR was performed with the high‐purity UCP multiplex PCR master mix (Qiagen, Hilden, Germany) on 2 μL DNA and the following primers: Fwd = CCTACGGGNGGCWGCAG. Rev. = GACTACHVGGGTATCTAATCC. In a subsequent PCR, index sequences were added to the purified PCR product. Samples were pooled and sequenced on an Illumina MiSeq with v2 reagents and 2 × 250 bp paired‐end reads. A sequencing depth of 20.000 reads per sample was aimed for. Negative controls and non‐template controls were added for each library preparation and sequencing run.

#### 2.2.2 Data Analysis of the Microbiome

After sequencing, paired reads were merged by their 3′ ends, followed by clustering unique reads into OTUs using the usearch programme v11.0.667 and a 97% sequence identity threshold [21]. Only amplicon sequences that occur at least twice were considered. Each OTU was assigned a representative sequence, and all reads were then aligned against the representative OTU sequences for determining the relative abundance. OTUs containing less than 0.01% of all reads were filtered out. The representative sequences of each OTU were searched in the NCBI 16S‐Microbial and NT reference databases using NCBI BLAST v2.12 and assigned to the species with the best BLAST alignments (by bit score). OTUs with multiple best matches were assigned to the least common ancestor of all alignments with the same BLAST bit score. If no alignment was found for an OTU, or only alignments with a sequence identity (PID) < 97%, it was considered unclassified. Rarefaction analysis was done on the OTU tables, with an acceptance threshold of 95% of OTUs at half read depth. As the negative controls yielded substantially fewer sequencing reads than the patient samples (mean read count: 1081 vs. 43 382 reads), reagent‐derived contamination was considered unlikely to have a meaningful impact on the observed microbiome profiles. Consequently, no bioinformatic contaminant identification or removal procedures were applied to the patient samples.

Various key figures for the biodiversity of the microbiome were calculated from the OTUs and their relative frequencies. All classified and unclassified OTUs that were not recognized as human sequences by BLAST were used for this purpose. The alpha diversity A was defined as the number of OTUs. The Shannon diversity H was calculated from the relative frequency p_*x*_ = n_x_/Σ_*i*_ n_*i*_ of each OTU × with the number of reads n_x_: H = −Σp_*i*_. ln p_*i*_. H thus depended on both the alpha diversity and the relative distribution and increases accordingly with an increasing number of OTUs and a more uniform distribution. To account for the repeated measure framework and the hierarchical data structure, Linear Mixed Models (LMMs) were estimated. Based on these models, post hoc tests were performed to evaluate changes in alpha diversity and the Shannon index over time as well as differences between women and infants at each time point.

Alpha diversity and the Shannon index were used to assess the richness and evenness within individual samples. Beta diversity was calculated to compare taxonomic composition between samples and over time. For comparisons between women and infants, similarity at the genus level was quantified as the proportion of shared genera relative to the total number of genera found in each comparison pair following the Jaccard similarity index [22]. This similarity index was calculated between different timepoints for individual mothers or children, as well as between mother–child pairs at corresponding timepoints. To evaluate the significance of observed similarities, the mean values (M) for each comparison pair were tested against the mean average similarity across all possible pairings using a paired *t*‐test.

### 2.3 Statistical Methods

We described the demographic characteristics of the participants using absolute and relative frequencies, means, standard deviations (SD), medians and interquartile ranges (IQR) and using analysis of variance (ANOVA) calculations for repeated measures. Women's skin characteristics were analyzed by ANOVA calculations for repeated measures. Significant differences between the three visits were determined using a post hoc *t*‐test with Bonferroni correction. Infant's skin characteristics were tested by 2‐sided *t*‐tests for paired groups between Visit 2 and Visit 3.

Associations of skin parameters of mothers and infants and skin microbiome characteristics were described using Pearson's correlation coefficients (*r*), whereas *r*‐values from 0.3 to 0.5 were regarded as medium and values higher than 0.5 as large [23]. The statistical significance of estimated correlation coefficients was tested and corresponding *p*‐values were adjusted based on the Benjamini‐Hochberg procedure. Furthermore, paired *t*‐tests and nonparametric permutation tests (metric observations) were used to compare paired data.

### 2.4 Methods to Examine Interactions

The following analyses were performed: Skin microbiome characteristics of women at Visit 1/2 and infants at Visit 2/3 were correlated. Because of a possible association between mode of delivery and the skin microbiome measured by alpha‐diversity, comparisons were made for vaginal and caesarean delivery. Correlations were made for microbiome (alpha‐diversity and Shannon index) and antibiotic intake, vaginal seeding, type of newborn's nutrition, house pets, caregivers and gestational age at birth. We calculated correlations between microbiome alpha‐diversity and individual skin characteristics at different time points (study visits). The analysis allowed us to examine potential associations between the skin characteristics of women and their infants and the microbiome. Associations of the microbiome (Shannon index) between mothers and their infants were described using Pearson's correlation coefficients (*r*), whereas *r*‐values from 0.3 to 0.5 were regarded as medium and values higher than 0.5 as large [23].

Due to the descriptive character of the study, all statistical analyses were exploratory. All statistical methods were performed using IBM SPSS Statistics, Version 20.0.2.0 (20) and R version 4.4.2.

## 3 Results

### 3.1 Participants

We included 109 pregnant women from March 2021 to September 2023, with no screening failures. Ninety‐five mother‐infant pairs attended the second visit, and 100 mother‐infant pairs attended the third study visit. Eight women dropped out after the first visit, whereas six mother‐infant pairs missed the second visit, and one mother‐infant pair missed the third visit. Ultimately, 93 mother‐infant pairs completed all visits. Reasons for dropping out or missing a visit varied and included: loss of interest, unable to follow through due to high workload with a newborn, infant illness, hospitalization, relocation from Berlin, extreme heat or fathers withholding consent.

### 3.2 Descriptive Data of Study Participants

Demographic and individual characteristics of included women are shown in Table 1.

**TABLE 1:** Characteristics of study participants.

	Women			Infants	
	Visit 1 (*n* = 109)	Visit 2 (*n* = 95)	Visit 3 (*n* = 100)	Visit 2 (*n* = 95)	Visit 3 (*n* = 100)
Age (year women/weeks infants), mean (SD)	33.05 (4.2)			4.06 (0.40)	25.6 (2.02)
BMI (kg/m^2^), mean (SD)	25.6 (4.0)	23.9 (3.9)	23.1 (4.05)		
Habitation Urban, *n* (%)	104 (95.4)			92 (96.8)	97 (89)
Taking antibiotics, *n* (%)	3 (2.8)	14 (14.7)	4 (4.0)		
Atopic predisposition, *n* (%)	3 (2.8)				
Dermatological findings (yes/no) (%)	13.9/86.1				
Medical history (yes) %	47.2				
Course of delivery (vaginal/caesarean birth/no information) (*n*)		70 vaginal births, 25 caesarean births			
Participants who applied skin products 24 h before study visits (*n*)	2	0	1	0	1
Sex of infant, female *n* (%)				39 (41.1)	
Sex of infant, male *n* (%)				56 (58.9)	
Gestational age at birth (weeks) mean (SD)				39.9 (1.14)	
Infant's weight (g) mean (SD)				4238 (622)	7955.5 (977)
Infant's height (cm) mean (SD)				54.3 (2.55)	68.1 (3.20)
Kind of nutrition: Breastfeeding *n* (%)				80 (84.2)	40 (40)
Kind of nutrition: infant formula feeding *n* (%)				2 (2.1)	6 (6)
Kind of nutrition: combination *n* (%)				13 (13.5)	54 (54)
Period of nutrition (weeks), mean (SD)				3.9 (0.76)	18.4 (9.85)
Eczema Area and Severity Index (EASI), mean (SD)				0.15 (0.48)	0.21 (0.49)
Caregivers (grandparents, nursery nurse, others) *n*				19	23
Pets (cats, dogs, others) *n*				23	21
Environment (apartment/house) *n*				86/6	93/7

Women's mean age was 33 (SD 4.2) (range 22–45) years. Mean BMI was 25.6 (SD 4.0) (range 18.7–41.2) kg/m^2^. Most of the women lived in an urban setting (95.4%); only three lived in a rural setting, and in two cases we had no information.

Demographic and individual characteristics of included infants are shown in Table 1. The infant cohort at V2 (4 weeks postpartum) included 95 participants. Infants were born at a mean gestational age of 39.9 weeks (SD 1.14). At V2 the mean weight of the infants was 4238 g (SD 622.1) (range 2860–6100 g), and the mean height was 54.3 cm (SD 2.5) (range 48–60 cm).

The infant cohort at V3 (6 months postpartum) included 100 subjects. The mean age at the third study visit of the infants was 25.6 weeks (SD 2.02), the mean weight was 7955.5 g (SD 977.07) and the mean height of the infants was 68.1 cm (SD 3.20). The mean period of breastfeeding was 18.4 weeks (SD 9.85). Forty infants (36.7%) were exclusively breastfed, 6 (5.5%) received only formula feeding, and 54 infants (49.5%) received a combination of both breastmilk and formula feeding.

At each scheduled study visit, a skin swab for microbiome analysis was collected from each participant, resulting in a total of 499 skin swabs.

### 3.3 Skin Function, Skin Structure

Skin functional and structural parameters of women and infants are shown in Table 2. Additional results of the statistical tests showing the post hoc tests can be found in Appendix S1 for Table 2 (Tables S5–S13).

**TABLE 2:** Skin function and skin structure in women and infants at Visit 1, Visit 2, and Visit 3.

	Women			Infants		
	Mean	SD	*n*	Mean	SD	*n*
TEWL V1 g/m^2^/h	9.53	0.19	93	n.a.	n.a.	n.a.
TEWL V2 g/m^2^/h	11.38^a^	6.44	93	11.25	6.24	94
TEWL V3 g/m^2^/h	13.80^b^	7.43	93	12.04	6.58	94
SCH V1	31.67	9.81	93	n.a.	n.a.	n.a.
SCH V2	33.49	8.65	93	36.88	11.50	94
SCH V3	38.59^b^, ^c^	10.29	93	41.24^d^	11.76	94
pH‐value V1	5.30	0.51	92	n.a.	n.a.	n.a.
pH‐value V2	5.13^a^	0.52	92	4.86	0.37	93
pH‐value V3	5.02^b^	0.49	92	4.99^d^	0.38	93
Epidermal thickness V1 μm	110.48	13.83	91	n.a.	n.a.	n.a.
Epidermal thickness V2 μm	110.37	14.57	91	129.46	13.67	80
Epidermal thickness V3 μm	107.66	9.75	91	129.74	13.21	80
Skin roughness RA V1 μm	9.65	2.20	67	n.a.	n.a.	n.a.
Skin roughness RA V2 μm	10.17	2.08	67	12.03	2.36	74
Skin roughness RA V3 μm	11.09^b^, ^c^	2.24	67	11.84	2.55	74
Skin roughness RZ V1 μm	39.79	8.83	67	n.a.	n.a.	n.a.
Skin roughness RZ V2 μm	39.68	7.99	67	43.72	10.23	74
Skin roughness RZ V3 μm	44.65^b^, ^c^	8.67	67	41.50	8.47	74
Skin stiffness V1 Uf, mm	0.31	0.06	92	n.a.	n.a.	n.a.
Skin stiffness V2 Uf, mm	0.31	0.06	92	0.29	0.09	92
Skin stiffness V3 Uf, mm	0.28^b^, ^c^	0.06	92	0.27	0.07	92
Skin elasticity V1 Ur/Uf %	0.79	0.06	92	n.a.	n.a.	n.a.
Skin elasticity V2 Ur/Uf %	0.75^a^	0.13	92	0.56	0.13	92
Skin elasticity V3 Ur/Uf %	0.68^b^, ^c^	0.14	92	0.62	0.15^d^	92

*Note:* Analysed by ANOVA calculations for repeated measures and tested for significance between visits by post hoc *t*‐test with Bonferroni‐correction in women. Analysed by *t*‐test for paired samples in infants.

Abbreviations: n.a., not applicable; V1, Visit 1; V2, Visit 2; V3, Visit 3.

^a^ Statistically significant difference between V1‐V2 in women.

^b^ Statistically significant difference between V1–V3 in women.

^c^ Statistically significant difference between V2‐V3 in women.

^d^ Statistically significant difference between V2 and V3 in infants.

### 3.4 Skin Microbiome of Women and Infants Over Time

Microbiome sequencing returned on average 43 382 reads per sample. The 18 negative controls/non‐template controls yielded 1081 reads on average. The top 5 genera found in the controls are listed in Table S14 Negative_controls_top5_taxa as well as corresponding OTU‐tables (the OTU‐tables can be found in the Data S1 ZIP‐File M OTU‐tables). Rarefaction analysis showed that all samples retained > 95% of OTUs at half read depth.

The taxonomic analysis across all samples, including mothers and infants, across all sampling times revealed bacteria from 30 different phyla, 125 different orders and 1217 different genera.

The five most prevalent bacterial phyla in women over all three visits according to their abundance were *Bacillota, Actinomycetota, Pseudomonadota, Bacteroidota* and *Fusobacteriota*. The five most prevalent bacterial phyla in infants over both visits according to their abundance were *Bacillota*, *Pseudomonadota*, *Actinomycetota*, *Bacteroidota* and *Fusobacteriota*. At phyla level, *Bacillota* was consistently dominant in both women and infants, followed by *Actinomycetota* in women and *Pseudomonadota* in infants.

**FIGURE 1:**
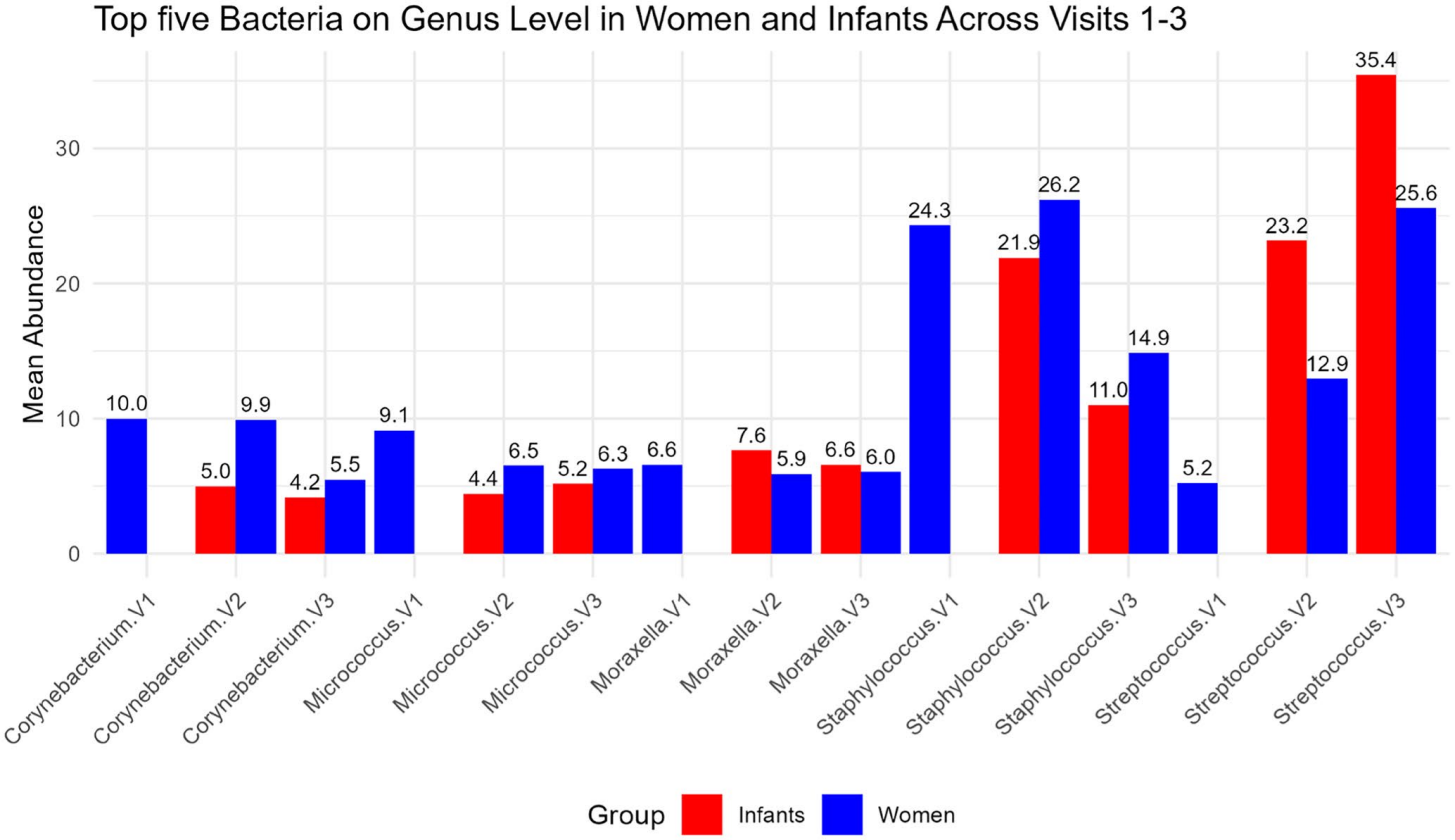
Mean values of top five most common bacteria on genus level for women and infants across Visits 1–3.

**FIGURE 2:**
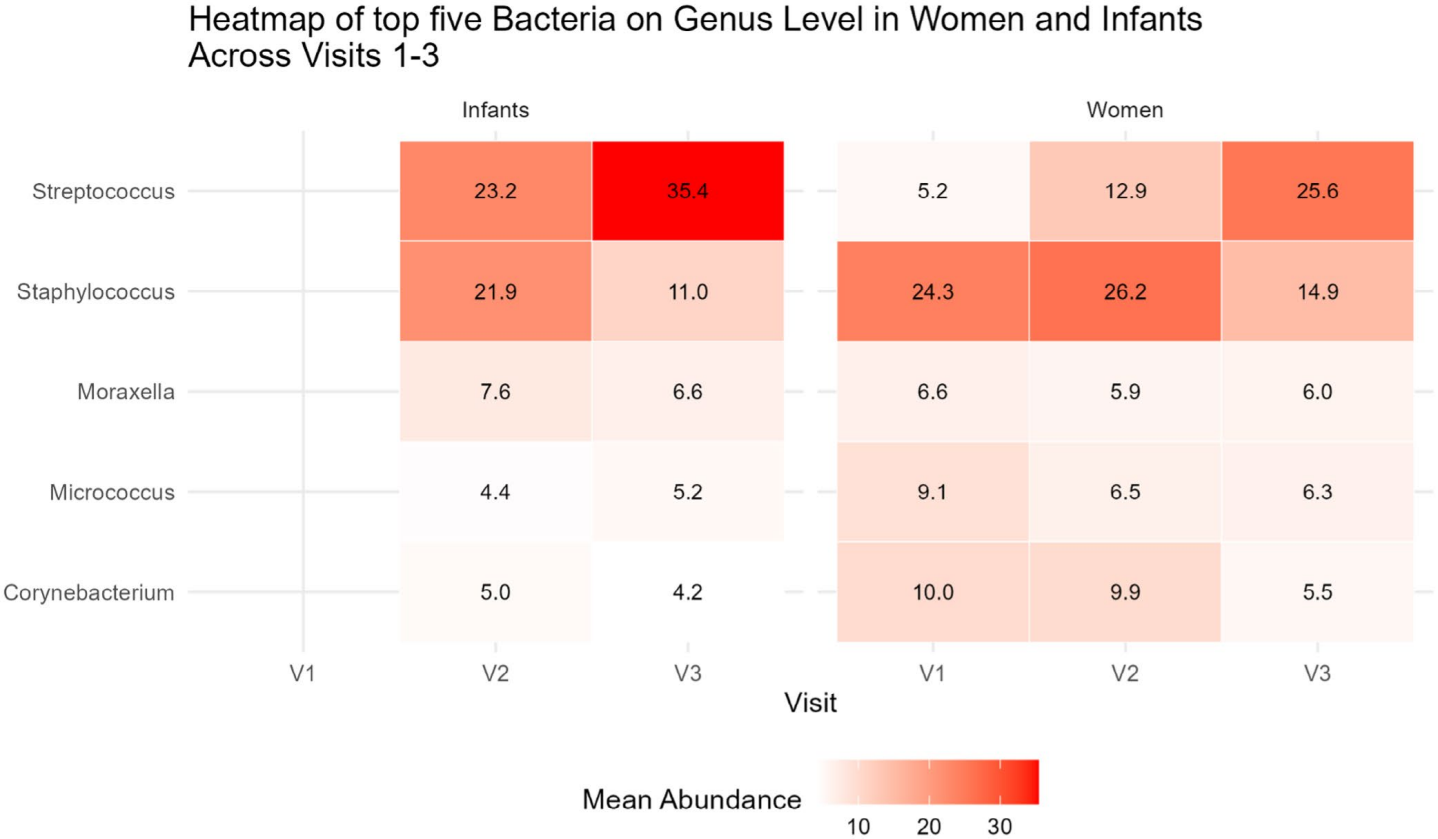
Heatmap of top five most common bacteria on genus level for women and infants across Visits 1–3.

The top five genera are demonstrated in Figure 1 according to their mean values and additionally displayed in Figure 2 which shows these data as a heatmap for an alternative representation.

The microbial diversity of both infants and women changed over the study period. Infant alpha diversity (observed species richness) increased substantially from 82.6 at Visit 2 to 116.1 at Visit 3 (Figure S1), representing a 40.6% rise. In contrast, women's alpha diversity showed a more gradual increase from 133.0 at Visit 1 (during pregnancy) to 145.4 at Visit 3 (6 months postpartum). To evaluate the described shifts in alpha diversity while simultaneously accounting for the repeated measures and the dependency within mother‐infant pairs, a LMM was estimated with group and time as fixed effects and family as a random effect. Post hoc tests based on the estimated LMM confirm that the observed change in alpha diversity of infants between Visit 2 and 3 is statistically significant (Δ = 33.1, SE = 10.6, CI = [12.2, 54], *p* < 0.01), whereas no statistically significant changes were found for women across all three pairwise comparisons. In addition, alpha diversity differed significantly between women and infants at Visit 2 (Δ = 37.5, SE = 10.8, CI = [16.3, 58.7], *p* < 0.001) and Visit 3 (Δ = 29.3, SE = 10.5, CI = [8.7, 49.9], *p* < 0.01). These findings were confirmed by nonparametric tests for longitudinal data, specifically a Wald‐type and ANOVA‐type rank‐based tests yielding consistent results.

Interestingly, while species richness changed, the Shannon diversity index, which accounts for both abundance and evenness, remained stable in infants between Visit 2 (4 weeks postpartum) and 3 (6 months postpartum). For women, the Shannon index showed slight fluctuation, decreasing from 2.97 at Visit 1 to 2.78 at Visit 2 before returning to near‐baseline levels (2.96) at Visit 3 (See Figure S2). As above, a LMM was estimated, this time using the Shannon index as the dependent variable. Post hoc analyses suggest no significant changes in the Shannon index for infants or women between visits. In contrast, we observe a significant difference in the Shannon index between mothers and infants at Visit 2 (Δ = 0.25, SE = 0.09, CI = [0.075, 0.42], *p* < 0.01) and Visit 3 (Δ = 0.36, SE = 0.09, CI = [0.19, 0.53], *p* < 0.001). These results were again corroborated by the same nonparametric rank‐based tests.

At the genus level, significant shifts were observed in the women's skin microbiome across visits. *Staphylococcus* dominated at Visits 1 (24.3%) and 2 (26.2%) but decreased substantially by Visit 3 (14.9%). Whereas *Streptococcus* showed a huge increase from 5.2% at Visit 1 to 25.6% by Visit 3, becoming the most abundant genus. *Corynebacterium* exhibited a notable decline across visits (10.0%, 9.9% and 5.5%, respectively). The community became increasingly diverse at Visit 3, with 18 genera exceeding the 1% relative abundance threshold compared to 14 at Visit 1 and 13 at Visit 2. Notably, genera such as *Veillonella*, *Rothia*, *Haemophilus* and *Neisseria* emerged above the 1% threshold by Visit 3.

The infant skin microbiome at the genus level was dominated by *Streptococcus* and *Staphylococcus* across both timepoints. Notably, *Streptococcus* increased substantially from 23.2% at Visit 2 to 35.4% at Visit 3, while *Staphylococcus* decreased by half (21.9%–11.0%). This pattern paralleled the shift observed in women's microbiota by Visit 3. Other abundant genera maintained relatively stable proportions between visits, including *Moraxella* (7.6%–6.6%) and *Micrococcus* (4.4%–5.2%). Visit 3 showed increased relative abundance of *Veillonella* (from 1.2% to 3.5%), *Bifidobacterium* (from 2.0% to 2.8%) and the emergence of *Rothia* (2.8%) and *Granulicatella* (1.7%) above the 1% threshold. Both visits had similar taxonomic diversity, with 14–15 genera exceeding the 1% abundance threshold.

### 3.5 Correlations Between Maternal and Infant Skin Microbiome

The Shannon diversity indices of mothers and infants demonstrated a moderate correlation at Visit 2 (*r* = 0.38, CI = [0.18, 0.54], *p* < 0.001), but this correlation was not present at Visit 3 (*r* = 0.08, CI = [−0.012, 0.28], *p* = 0.410). The *p*‐values were adjusted using the Benjamini–Hochberg procedure. The significance did not change for each coefficient based on Pearson and Spearman correlations.

**FIGURE 3:**
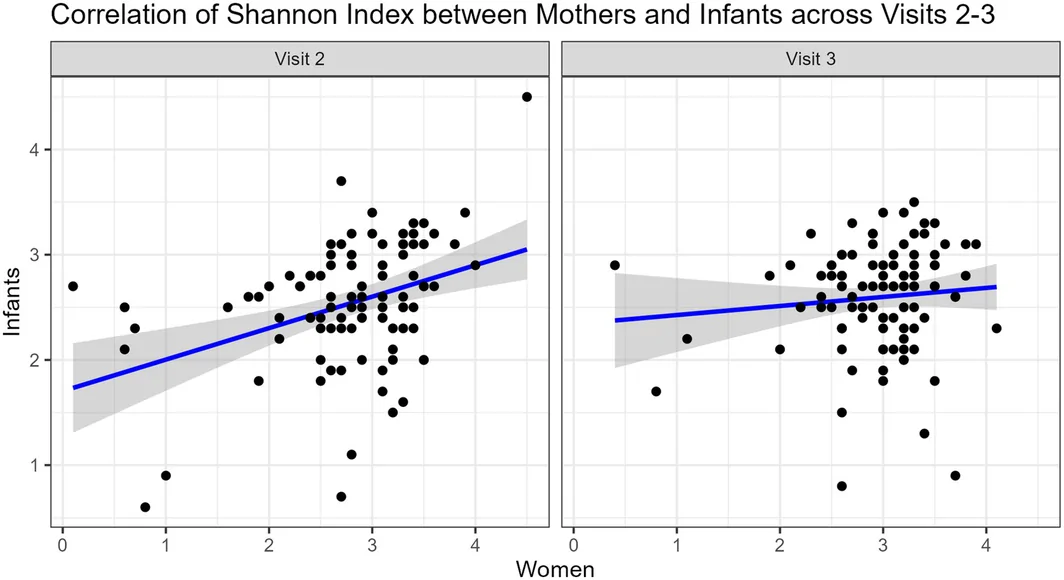
Scatterplots and OLS regression line with estimated 95% confidence intervals for the Shannon index across Visits 2–3 for women and infants.

Figure 3 displays scatterplots of the Shannon index as well as regression lines estimated using ordinary least squares (OLS) with 95% confidence intervals differentiated by Visit 2 and 3.

### 3.6 Beta‐Diversity Analysis of Maternal and Infant Skin Microbiome

#### 3.6.1 Longitudinal Changes in Maternal Microbiome

This within‐subject similarity was significantly higher than the between‐subject similarity (M = 0.04, SD = 0.12) when comparing a woman's Visit 1 microbiome to other women's Visit 2 microbiomes (*t*(92) = 2.57, Δ = 0.04, CI = [0.008, 0.065], *p* < 0.0119, Cohen's d = 0.26). The corresponding data are displayed in Figure S3, which shows a comparison of within‐ and between‐subject similarity based on beta diversity for women and infants across visits.

In contrast, the within‐subject similarity between Visit 2 and Visit 3 (6 months postpartum) was not significantly higher than the between‐subject similarity when comparing a woman's Visit 2 microbiome to other women's Visit 3 microbiomes (*t*(92) = 0.97, Δ = 0.010, CI = [−0.010, 0.034], *p* = 0.33, Cohen's d = 0.1). Both test results are confirmed by a nonparametric permutation test (*p* = 0.01, *p* = 0.34).

#### 3.6.2 Longitudinal Changes in Infant Microbiome

The microbiome similarity between infants at 4 weeks (V2) and 6 months (V3) after birth is M = 0.57 (SD 0.12). This within‐subject similarity was significantly higher than the between‐subject similarity (M = 0.55, SD = 0.1) when comparing an infant's Visit 2 microbiome to other infants' Visit 3 microbiomes (*t*(89) = 2.04, Δ = 0.013, CI = [0.000, 0.026], *p* = 0.044, Cohen's d = 0.21). A nonparametric permutation test confirms this result (*p* = 0.04).

#### 3.6.3 Mother‐ Infant Microbiome Similarities

The microbiome similarity between a mother (V2) and her own infant (V2) at 4 weeks postpartum averaged M = 0.61 (SD 0.14). This mother‐infant pair similarity was significantly higher than the between‐pair similarity (M = 0.54, SD = 0.08) when comparing a mother's microbiome to unrelated infants (*t*(91) = 6.72, Δ = 0.077, CI = [0.054, 0.1], *p* < 0.001, Cohen's d = 0.69). A nonparametric permutation test corroborates this result (*p* < 0.001).

**FIGURE 4:**
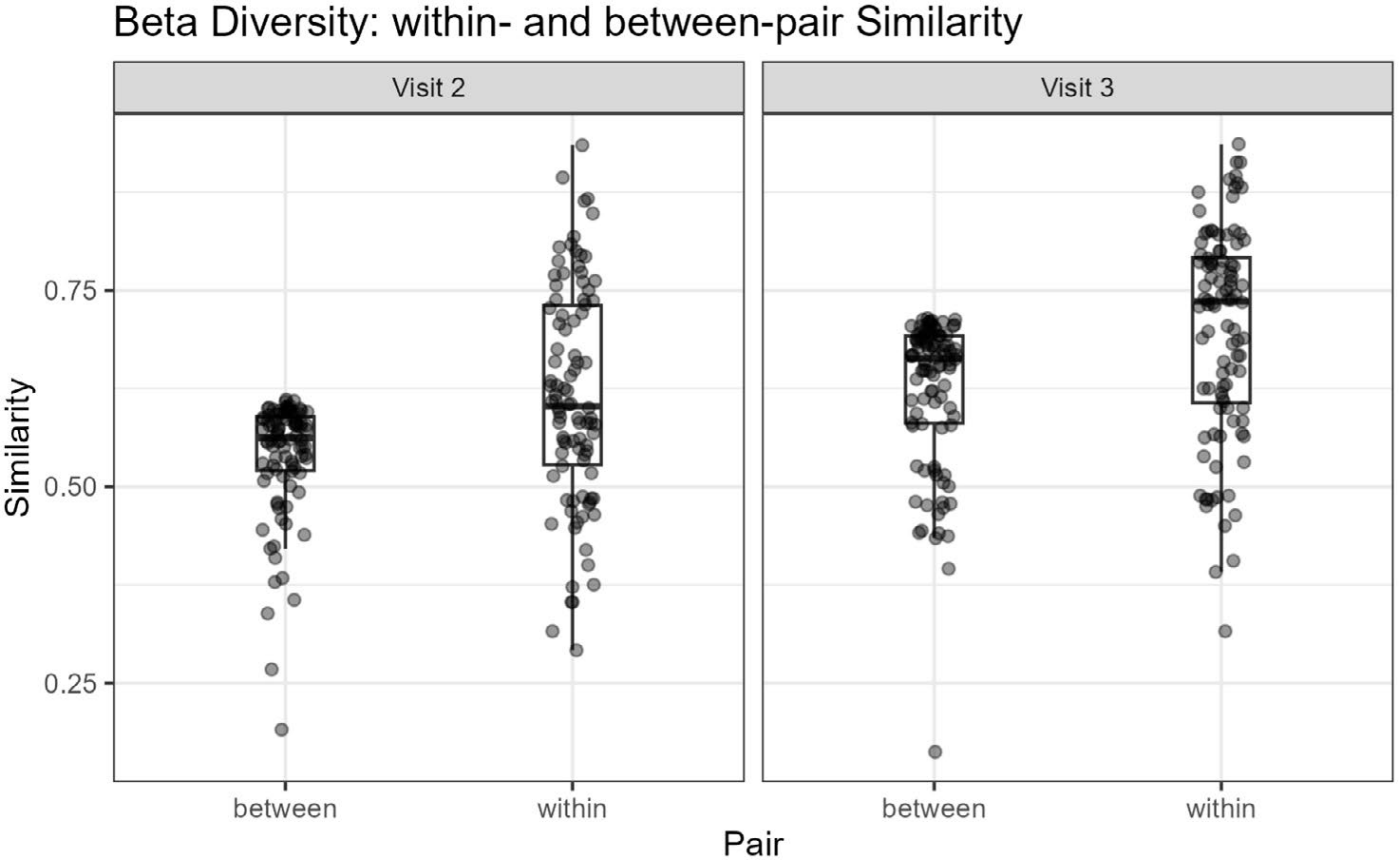
Boxplots of beta diversity for within‐ and between‐pair similarity across visits.

This effect is also illustrated in Figure 4, which shows the distribution of similarity based on beta diversity for dyadic versus random pairings across Visits 2 and 3 using boxplots. At 6 months postpartum, the mother‐infant pair similarity (M = 0.7, SD = 0.13) remained significantly higher than the between‐pair similarity (M = 0.62, SD = 0.1) when comparing a mother's microbiome to unrelated infants (*t*(91) = 8, Δ = 0.07, CI = [0.057, 0.095], *p* < 0.001, Cohen's d = 0.8). Again, a nonparametric permutation test confirms this finding (*p* < 0.001).

The analysis of within‐ and between‐pair similarity was repeated for male versus female infants as well as caesarean versus vaginal delivery. In all four subgroups, both the paired *t*‐test and its nonparametric permutation version confirmed the results stated above: the within‐pair similarity is always significantly higher than between‐pair similarity for both time points (all *p* < 0.001).

#### 3.6.4 Correlation Between Microbiome and Individual or Environmental Characteristics

We found no significant correlations between diversity metrics (Alpha diversity and Shannon index) and any individual or environmental characteristics, for either maternal or infant microbiomes across all visits (see Table S2). Mode of delivery showed no correlation with infant microbiome diversity measures (alpha diversity and Shannon index).

#### 3.6.5 Correlation Between Microbiome Diversity and Skin Characteristics

The correlation between maternal alpha diversity at V1 and V2 and infant skin characteristics at V2 was weak, ranging from −0.298 to 0.288 (Table S3 Correlation between maternal microbiome (V1 and V2) and infant skin characteristics (V2)). Furthermore, correlations between maternal microbiome diversity at V2 and V3 and infant skin characteristics at V3 ranged from −0.306 to 0.133 (Table S4 Correlation between maternal microbiome (V2 and V3) and infant skin characteristics (V3)). While a moderate negative correlation (−0.306) was observed between infant skin stiffness at V3 and maternal alpha diversity at V3, no correlation was established between these variables. For all estimated correlation coefficients, the estimated *p*‐values were simultaneously adjusted utilizing the Benjamini‐Hochberg procedure yielding no statistically significant estimates for a significance level of 0.05.

## 4 Discussion

The skin microbiome of the infants and their mothers showed a taxonomic overlap (*r* = 0.38, CI = [0.18, 0.54], *p* < 0.001 at 4 weeks), which diminishes by 6 months (*r* = 0.01, CI = [−0.9, 0.29], *p* = 0.324), aligning with the hypothesis of early maternal microbiota imprinting followed by diversification. Both groups shared the same five predominant phyla: *Bacillota*, *Actinomycetota*, *Pseudomonadota*, *Bacteroidota* and *Fusobacteriota*. *Bacillota* dominated across all cohorts (ranging from 41% to 57%). The second most abundant phylum differed between cohorts: *Actinomycetota* in mothers across all visits versus *Pseudomonadota* in infants.

Our findings partially align with previous investigations of the human skin microbiome. Grice et al., who first provided large scale results of the skin microbiome in healthy adults, identified the same major phyla were, but in different proportions: *Actinomycetota* (51.8%) (*Actinobacteria* formally known), *Bacillota* (24.4%) (*Firmicutes* formally known), *Pseudomonadota* (16.5%), (*Proteobacteria* formally known) and *Bacteroidota* (6.3%) (*Bacteroidetes* formally known) [2]. Similarly, Capone et al. showed that infant skin (up to 12 months of age) was predominantly colonized by *Bacillota* (*Firmicutes* formally known), followed in abundance by *Actinomycetota* (*Actinobacteria* formally known), *Pseudomonadota* (*Proteobacteria* formally known) and *Bacteroidota* (*Bacteriodetes* formally known) [8]. This taxonomic distribution is similar to our infant results, with the exception that *Pseudomonadota* ranked as the second most abundant phylum in our infant cohort, while it was the third most abundant phylum in Capone et al. study.

The volar forearm, categorized as a dry skin site, typically exhibits high microbial diversity [2]. Consistent with this classification, we observed a mixed community dominated by *Cutibacterium*, *Staphylococcus*, *Corynebacterium* and *Streptococcus* species across all measurement timepoints in our female participants. This composition pattern remained stable throughout the study period and corresponds with the expected microbiome profile for this anatomical region [3].

Oh et al. showed a temporal stability in adults' skin microbiome [4]. The alpha diversity and the Shannon index of the women's microbiome reduced slightly from pregnancy to 4 weeks after birth and then got richer again by 6 months postpartum. The larger effect size for the pregnancy‐to‐early postpartum comparison (Visit 1 and 2) versus the early‐to‐late postpartum (Visit 2 and 3) comparison indicates that the immediate postpartum period may represent a more substantial shift in the maternal skin microbiome than later postpartum changes.

The infant's microbiome changed during the study period; even though it became more diverse by 6 months postpartum, it did not reach the level of maternal diversity. Our findings suggest an initial maternal influence on infant skin microbiota that diminishes over time. The moderate correlation in Shannon diversity indices at Visit 2 disappearing by Visit 3 suggests that infants develop more individualized diversity profiles as they age.

Gaitanis et al. reported similar results of an increasing skin flora of growing infants, with several cultured genera per individual peaking for both mothers and infants in the period sixth to ninth month postpartum [12]. They also showed that the rates of concordant isolation of the same microbial species within the mother‐infant dyad tended to drop from birth to the end of the first year postpartum [12]. These patterns could reflect the combined effects of reduced physical contact, increasing environmental exposures and developmental changes in infant skin physiology with age, besides the mother‐infant transmission.

Still, our analysis reveals a striking degree of microbiome similarity between mothers and their own infants that exceeds random mother‐infant pairings. This strong mother‐infant microbiome association persists and even strengthens from 4 weeks to 6 months postpartum, as evidenced by the increasing similarity values (0.61–0.70) and effect sizes (0.69–0.80). Furthermore, the effect sizes support this mother‐infant microbiome association between mother‐infant pairs. The strengthening of this association over time could suggest an ongoing microbial interaction between mothers and their infants, even though the present observational 16S amplicon design cannot distinguish direct transmission from shared environmental or household influences.

The beta diversity analysis of infant skin microbiomes reveals significant individual‐specific signatures that persist through early development. Despite the considerable physiological and environmental changes occurring during the first 6 months of life, each infant's microbiome maintains greater similarity to its earlier state than to other infants' microbiomes. The low effect size (Cohen's d = 0.21) indicates individual persistence in microbiome composition. Interestingly, the within‐subject similarity value for infants (0.57) parallels that observed in‐maternal‐samples across visits, suggesting similar levels of temporal stability in both populations despite the more dynamic developmental state of infants. This finding suggests that early colonization patterns may establish relatively stable individual‐specific communities that persist despite ongoing development.

Chu et al. showed that while the microbial composition was initially influenced by mode of delivery at birth, these differences disappeared by 6 weeks of age, when the microbiome reorganization was primarily based on body site rather than delivery mode [7]. This is in accordance with our study results, which found no correlations between microbiome diversity and mode of delivery.

The absence of significant correlations between microbiome diversity and individual or environmental factors suggests that skin bacterial community structure may be influenced by more complex interactions than single variables alone. Particularly notable is the lack of association between delivery mode and infant microbiome diversity, contrasting with findings often reported for gut microbiome studies [24]. The weak correlations between maternal microbiome diversity and infant skin properties indicate that the relationship between diversity metrics and skin physiology is more nuanced.

These findings highlight the complex relationship between microbial diversity and host phenotype, suggesting that functional rather than taxonomic profiling might reveal more meaningful associations in future studies.

### 4.1 Generalisability

In our study we included a convenient sample of eligible women during pregnancy in the city of Berlin by advertising strategies that included: the departments website and flyers in outpatient services of gynaecologist/obstetrician. Regarding age, women in our study had a mean age of 33 years, higher than the German average of 30.1 years for first‐time mothers in Germany [25]. This difference may be because not all women included were first‐time mothers, which contributed to a higher mean age in our sample.

Regarding the mean birth weight and gestational age at birth, our findings were representative for the infant population in Germany [26]. When examining the composition of the skin microbiome, one limitation might be that we predominantly included women and infants from an urban setting. This raises the possibility that people living in a rural setting might have different bacterial compositions. Thus, our study results might be restricted to pregnant women and infants from 4 weeks to 6 months of age.

### 4.2 Limitations

Our very low dropout rate suggests no potential for attrition bias. The study specifically tracks potentially relevant microbial sources: the environment (urban or rural setting), the included caregivers, pets in the family, taking of antibiotics or course of delivery. The participants in the study were selected by convenience sampling method mostly in an urban setting. So potentially, there might be an influence in the composition of the microbiome due to habitation. But probably, other relevant factors such as ethnicity, which may have contributed to the infant's skin colonization, are possible and should be investigated in further studies. While these factors might introduce variability, the study's standardized sampling methods, strict inclusion criteria and high participant retention rate contribute to the robustness of the core findings, even though we only used a single‐site sampling design. This was done for practical reasons, to ensure that swab sampling was feasible in a paediatric setting. Therefore, these limitations are unlikely to significantly compromise the reliability of the conclusions regarding maternal–infant microbiome concordance and developmental dynamics. In addition, the microbiome data were analysed using an OTU‐based rather than an ASV‐based workflow. Although ASV‐based approaches may provide higher sequence resolution in some settings, OTU‐based clustering can reduce the impact of over‐splitting and was considered appropriate for the objectives of the present study, which focused primarily on community composition at higher taxonomic levels rather than species‐level resolution. However, regarding the OTU‐based approach, we have to state that V3–V4 16S sequencing has limited species‐ and strain‐level resolution and that residual contamination cannot be fully excluded in these low‐biomass skin samples. Furthermore, the use of an OTU‐based approach may limit direct comparability with studies employing ASV‐based pipelines and should be considered when interpreting the findings.

### 4.3 What This Study Can/Cannot Infer

- While the maternal microbiome revealed only minor changes from pregnancy to 6 months postpartum, the infant microbiome showed increasing diversity between 4 weeks and 6 months of age, albeit not yet reaching the level of diversity of the maternal skin microbiome.
- Regarding the microbiome consistency of the included women and infants, the within‐pair similarity was always significantly higher than between‐pair similarity for both time points.
- Due to the descriptive character of the observational study, no causal conclusions can be driven.
- The absence of significant correlations between microbiome diversity and individual or environmental factors suggests that skin bacterial community structure may be influenced by more complex interactions than single variables alone. Particularly notable is the lack of association between delivery mode and infant microbiome diversity, contrasting with findings often reported for gut microbiome studies [24]. The weak correlations between maternal microbiome diversity and infant skin properties indicate that the relationship between diversity metrics and skin physiology is more nuanced.

## 5 Conclusion

We performed a prospective cohort study examining the skin bacterial microbiome in mother‐infant pairs from pregnancy through 6 months postpartum. Maternal skin microbiome revealed mild to moderate changes during pregnancy and postpartum periods. Infant microbiome showed increasing diversity between 4 weeks and 6 months of age, albeit not yet reaching the level of diversity of the maternal skin microbiome. Regarding the microbiome consistency of the included women and infants, the within‐pair similarity was always significantly higher than between‐pair similarity for both time points. Therefore, we conclude that there is likely a relationship between the microbiome of the mother and that of her child. No associations between environmental (e.g., house pets, caregivers) or individual characteristics (e.g., taking of antibiotics, course of delivery) nor skin characteristics and the microbiome were detected.

### 5.1 Implications for Further Research

This prospective cohort study provided evidence of the skin microbiome in healthy mothers and their healthy infants over pregnancy up to 6 months postpartum with mild to moderate changes during pregnancy. In our cohort, women with clinically no signs of skin affections or dermatitis have been included.

Based on these results, the following research questions can be addressed and warrant further investigations:

1. To investigate skin care regimen and its influence on skin microbiome during pregnancy and during the period after birth.
2. To examine the relationship between maternal and infant microbiomes, when at least one of them has an impaired skin barrier, and to determine whether restoring the microbiome helps improve skin barrier function.
3. To investigate the molecular mechanisms across different skin layers, along with the characteristics of the skin microbiome, to better understand how they interact.

## Author Contributions

U.B.‐P.: Conceptualization, funding acquisition, methodology, resources, supervision, writing – review and editing. J.K.: Conceptualization, methodology, writing – review and editing. D.W.: Project administration, investigation, writing – original draft. A.C.: Investigation, writing – original draft, review and editing. A.F.: Investigation, writing – review and editing. R.S.: Formal analysis, writing – original draft, review and editing. P.M.: Formal analysis, writing – original draft, review and editing. G.Z.: Conceptualization, review and editing. K.H.: Investigation, writing – review and editing. T.T.‐S.: Investigation, writing – review and editing. R.A.: Investigation, writing – review and editing. G.E.: Formal analysis, writing – review and editing. J.R.: Formal analysis, writing – review and editing. F.K.: Formal analysis, writing – review and editing.

## Funding

This observational study is an investigator‐initiated trial conducted at the Charité–Universitätsmedizin Berlin. Germany. The project was financially supported by the Dehaa Rossun Research Center of Lunaler Group, Kowloon, Hong Kong. This funding organization was included in the development of the study concept but was not included in the analysis, Interpretation and publication of the results of the study. The funder had no role in the data collection, data analysis and reporting of this study.

## Ethics Statement

The study was conducted in accordance with the Declaration of Helsinki and received approval from the Ethics Committee of the Charité–Universitätsmedizin Berlin (reference number is EA2/184/20) on December 14, 2020.

## Consent

Written informed consent was obtained from the included women. For the infants, written informed consent was obtained by both parents or from the parent with sole custody.

## Conflicts of Interest

Ulrike Blume‐Peytavi is a scientific member of the Board of the Dehaa Rossun Research Center. Gavin Zhou is head of the Dehaa Rossun Research Center of Lunaler Group, Hong Kong. All other authors state that they have no conflicts of interest.

## Supporting information


**Figure S1:** Alpha Diversity in Women and Infants across Visits 1–3.
**Figure S2:** Boxplots of Shannon Index in Women and Infants across Visits 1–3.
**Figure S3:** Boxplots of similarity based on Beta diversity for within‐ and between‐subject similarity across visits for women and infants.
**Table S1:** Study variables.
**Table S2:** Correlation_Individual_External factors and Microbiome.
**Table S3:** Correlation between maternal microbiome (V1 and V2) and infant skin characteristics (V2).
**Table S4:** Correlation between maternal microbiome (V2 and V3) and infant skin characteristics (V3).
**Tables S5–S13:** Eight tables pairwise comparison in skin characteristics in women.
**Table S14:** Negative controls Top5 Taxa.

**Data S1:** exd70349‐sup‐0002‐DataS1.zip.

## Data Availability

The data that support the findings of this study are available on request from the corresponding author. The data are not publicly available due to privacy or ethical restrictions.
