# Supplementary material for: Maternal and Infant Skin Microbiome Synchrony and Divergence: A Longitudinal Study of Skin Microbial Ecology and Early‐Life Assembly
Source: Exp Dermatol. 2026 Aug 21;35(8):e70349. doi: 10.1111/exd.70349 (PMC13498444; doi:10.1111/exd.70349)
Supplement: Supplementary file 1 [file EXD-35-e70349-s001.zip › Suppl_ Material_Tables_A_D_Figures_A-C.docx]

**Supplementary material: Figures 1-3; Tables 1-4**

Figure 1 Alpha Diversity in Women and Infants across Visits 1-3

Figure 2 Boxplots of Shannon Index in Women and Infants across Visits 1-3

Figure 3 Boxplots of similarity based on Beta diversity for within- and between-subject similarity across visits for women and infants.

Table 1 Study variables

| **Variables** | Statistics / level of scales | Possible range of measurements | Time of measurement women | Time of measurement infant | Source of information |
| --- | --- | --- | --- | --- | --- |
| **Age** | metric variable | in years women  in days/months infant  18-45 ys women  0-28 d infant  1-6 mo infant | Baseline t0 | Delivery +28 d t1  Delivery +6 mo t2 | ID-card woman  “U-Heft” infant  U3: week 4-5  U5: month  6-7 |
| **Weight** | metric variable | in kg woman  in g infant | Baseline t0  Delivery +28d t1  Delivery + 6mo t2 | Delivery +28 d t1  Delivery +6 mo t2 | Measurement  “U-Heft” infant  U3: week 4-5  U5: month  6-7 |
| **Height** | metric variable | in cm woman  in cm infant | Baseline t0 | Delivery +28 d t1  Delivery +6 mo t2 | self-reporting woman  “U-Heft” infant  U3: week 4-5  U5: month  6-7 |
| **Sex** |  | female  male |  | Delivery +28 d t1 | “U-heft” infant  U1 delivery |
| **Habitation** | nominal variable | urban  rural | Baseline t0  Delivery +28 d t1  Delivery  +6 mo t2 | Delivery +28 d t1  Delivery +6 mo t2 | self-reporting woman |
| **Infant’s environment** | nominal variable | apartment  house |  | Delivery +28 d t1  Delivery +6 mo t2 | self-reporting woman |
| **Course of delivery** | nominal variable | vaginal birth  Caesarean | Delivery +28 d t1 |  | “U heft” infant  U1 delivery |
| **Vaginal seeding** | nominal variable | Yes  No |  | Delivery +28 d t1 | self-reporting woman |
| **Infant nutrition** | nominal variable | breastfeeding  cow`s-milk based infant formula (including Hypoallergenic (HA) - based formula) feeding  or combination |  | Delivery +28 d t1  Delivery +6 mo t2 | self-reporting woman |
| **Period of Infant’s nutrition (supplementary feeding)** | metric variable | from week x to week x |  | Delivery +28d t1  Delivery +6 mo t2 | self-reporting woman |
| **Gestational age at birth** | metric variable | in weeks |  | Delivery +28 d t1 | “U-heft” infant  U1 delivery |
| **Dermatological skin assessment mother** | nominal variable | coded by ICD11 | Baseline t0  Delivery +28d t1  Delivery +6 mo t2 |  | Study MD |
| **Dermatological skin assessment infant** | -Eczema  -Dry skin  -Itchy skin  -Rash  -Flexural dermatitis and/or visible dermatitis  -Diaper dermatitis  -Hand. Mouth and Foot Disease | EASI-Score:  Erythema  Edema/Papulation  Excoriation  Lichenification  EASI-Score:  Severity of Signs  0-3  none-severe  Dry skin:  desquamation fissures/rhagades  erythema  pruritus  Itchy skin:  if yes: continuous/intermittend  rash: yes/no  Diaper dermatitis: yes/no  Hand. Mouth and Foot Disease: yes/no |  | Delivery +28d t1  Delivery +6 mo t2 | Study MD |
| **Skin care mother** | Open ended question |  | Baseline t0  Delivery +28d t1  Delivery +6 mo t2 |  | self-reporting woman |
| **Skin care infant** | Open ended question |  |  | Delivery +28d t1  Delivery +6 mo t2 | self-reporting woman |
| **Care givers at home** | nominal variable | father  sibling  grandparents  Au-pair  nursery nurse  other |  | Delivery +28d t1  Delivery +6 mo t2 | self-reporting woman |
| **Fur-pets at home** | nominal variable | cat  dog  others | Baseline t0 |  | self-reporting woman |
| **Atopic predisposition of the mother** | nominal variable | Yes/No  at least one parent or sibling with physician-diagnosed AD. asthma or allergic rhinitis/rhino conjunctivitis as reported by at least by one parent but in otherwise good overall health) | Baseline t0 |  | self-reporting woman |
| **Taking of antibiotics** | nominal variable | Yes/No | Baseline t0  Delivery +28d t1  Delivery + 6mo t2 |  | self-reporting woman |
| **Alcohol consumption** | nominal variable | Yes/No | Baseline t0  Delivery +28d t1  Delivery + 6mo t2 |  | self-reporting woman |
| **Skin surface topography** | metric scaled variable  Mean (SD) | RA: 0.0-100.0 µm  RZ: 0.0-100.0 µm  Roughness:  RA (arithmetic mean roughness) in µm  RZ (arithmetic mean roughness from five sampling length) in µm | Baseline t0  Delivery +28 d t1  Delivery + 6 mo t2 | Delivery + 28 d t1  Delivery +6 mo t2 | Standardized images via Visioscan VC 98 USB  Skin area: inner forearm |
| **Epidermal thickness** | metric scaled variable  Mean SD | 0.0-100.0 µm  (40-60 µm) | Baseline t0  Delivery +28 d t1  Delivery + 6 mo t2 | Delivery + 28 d t1  Delivery +6 mo t2 | Standardized images via Optical coherence tomography (OCT)  THORLABS TELESTO  Skin area: inner forearm |
| **Skin elasticity** | metric scaled variable  Mean (SD) | 0.00-0.50  total extensibility or stiffness (Uf/mm)  elastic recovery or firmness (Ur/Uf in %) | Baseline t0  Delivery +28 d t1  Delivery + 6 mo t2 | Delivery + 28 d t1  Delivery +6 mo t2 | Cutometer MPA 580  Skin area: inner forearm |
| **Transepidermal water loss (TEWL)** | metric scaled variable  Mean (SD) | 0.0-60.0  g/m^2^/h | Baseline t0  Delivery +28 d t1  Delivery + 6 mo t2 | Delivery + 28 d t1  Delivery +6 mo t2 | Tewameter 300  Skin area: inner forearm |
| **Skin surface pH** | metric scaled variable  Mean (SD) | >4 or <7.5  Concentration of hydrogen ions | Baseline t0  Delivery +28 d t1  Delivery + 6 mo t2 | Delivery + 28 d t1  Delivery +6 mo t2 | Skin pH-meter® PH 905  Skin area: inner forearm |
| **Stratum corneum hydration (SCH)** | metric scaled variable  Mean (SD) | 0-120. arbitrary units (AU). whereas higher readings indicate higher stratum corneum hydration  Capacitance measurement of hydration | Baseline t0  Delivery +28 d t1  Delivery + 6 mo t2 | Delivery + 28 d t1  Delivery +6 mo t2 | Corneometer CM 825  Skin area: inner forearm |
| **Overall dry skin score**  **ODS** | Ordinal scaled variable  Frequency and distribution | 0-4 for each body site  Head / Neck  Trunk  Upper extremities  Lower extremities |  | Delivery +28 d t1  Delivery + 6 mo t2 | Dermatological examination  Skin inspection |
| **Eczema Area severity index**  **EASI** | Metric scale  Mean / SD | Head / Neck  Trunk  Upper extremities  Lower extremities |  | Delivery +28 d t1  Delivery + 6 mo t2 | Dermatological examination  Skin inspection |

Table 2 Correlation matrix please see the separate Exel File in the Supplementary material

Table 3 Correlation between maternal microbiome (V1 and V2) and infant skin characteristics (V2)

|  |  | Women V1 | Women V2 | Infants V2 | | | | | | | |
| --- | --- | --- | --- | --- | --- | --- | --- | --- | --- | --- | --- |
|  |  | Alpha.M.V1 | Alpha.M.V2 | TEWL | SCH | pH | RA | RZ | Epidermal thickness | Skin stiffness | Skin elasticity |
| Women V1 | Alpha.M.V1 | 1 |  |  |  |  |  |  |  |  |  |
| Women V2 | Alpha.M.V2 | 0,159 | 1 |  |  |  |  |  |  |  |  |
| Infants V2 | TEWL | 0,142 | 0,12 | 1 |  |  |  |  |  |  |  |
|  | SCH | 0,092 | 0,288 | 0,365 | 1 |  |  |  |  |  |  |
|  | pH | -0,052 | -0,19 | -0,315 | -0,258 | 1 |  |  |  |  |  |
|  | RA | -0,023 | 0,112 | 0,249 | 0,157 | -0,169 | 1 |  |  |  |  |
|  | RZ | -0,043 | 0,127 | 0,194 | 0,062 | -0,168 | 0,87 | 1 |  |  |  |
|  | Epidermal  thickness | 0,111 | -0,07 | 0,181 | -0,029 | -0,15 | 0,19 | 0,235 | 1 |  |  |
|  | Skin stiffness | 0,021 | -0,298 | 0,125 | -0,025 | -0,196 | 0,027 | 0,134 | 0,019 | 1 |  |
|  | Skin elasticity | -0,121 | -0,249 | 0,081 | 0,023 | -0,173 | -0,149 | -0,155 | 0,073 | 0,505 | 1 |

Table 4 Correlation between maternal microbiome (V2 and V3) and infant skin characteristics (V3)

|  |  | Women V2 | Women V3 | Infants Visit 3 | | | | | | | |
| --- | --- | --- | --- | --- | --- | --- | --- | --- | --- | --- | --- |
|  |  | Alpha | Alpha | TEWL | SCH | pH | RA | RZ | Epidermal thickness | Skin stiffness | Skin elasticity |
| Women V2 | Alpha | 1 |  |  |  |  |  |  |  |  |  |
| Women V3 | Alpha | 0,05 | 1 |  |  |  |  |  |  |  |  |
| Infants V3 | TEWL | -0,037 | 0,051 | 1 |  |  |  |  |  |  |  |
|  | SCH | 0,088 | 0,065 | 0,442 | 1 |  |  |  |  |  |  |
|  | pH | 0,097 | 0,133 | -0,202 | -0,104 | 1 |  |  |  |  |  |
|  | RA | 0,251 | -0,002 | 0,046 | -0,121 | 0,04 | 1 |  |  |  |  |
|  | RZ | 0,25 | 0,097 | 0,084 | -0,104 | 0,129 | 0,828 | 1 |  |  |  |
|  | Epidermal thickness | -0,028 | 0,062 | -0,029 | -0,209 | 0,061 | 0,129 | 0,062 | 1 |  |  |
|  | Skin stiffness | -0,17 | -0,306 | -0,25 | -0,171 | -0,097 | -0,237 | -0,308 | -0,013 | 1 |  |
|  | Skin elasticity | -0,172 | -0,223 | -0,199 | -0,265 | -0,166 | -0,262 | -0,321 | -0,046 | 0,657 | 1 |
