# Supplementary material for: Maternal and Infant Skin Microbiome Synchrony and Divergence: A Longitudinal Study of Skin Microbial Ecology and Early‐Life Assembly
Source: Exp Dermatol. 2026 Aug 21;35(8):e70349. doi: 10.1111/exd.70349 (PMC13498444; doi:10.1111/exd.70349)
Supplement: Supplementary file 1 [file EXD-35-e70349-s001.zip › Suppl_Material_Tables_E-M.docx]

**Supplementary material for Table 2: Skin function and skin structure in women and infants at Visit 1, Visit 2, Visit3**

8 tables pairwise comparison in skin characteristics in women

**Tables 5-13**

Table 5

| **Pairwise comparison** | | | | | | |
| --- | --- | --- | --- | --- | --- | --- |
| Transepidermal waterloss TEWL | | | | | | |
| (I) tewl | (J) tewl | Mean diff.(I-J) | SE | Sig.^b^ | 95% Confidence interval  for diff^b^ | |
|  |  |  |  |  | Lower limit | Upper limit |
| V1 | V2 | -1,855^*^ | ,694 | ,027 | -3,547 | -,162 |
|  | V3 | -4,272^*^ | ,780 | <,001 | -6,174 | -2,370 |
| V2 | V1 | 1,855^*^ | ,694 | ,027 | ,162 | 3,547 |
|  | V3 | -2,417 | ,999 | ,052 | -4,853 | ,019 |
| V3 | V1 | 4,272^*^ | ,780 | <,001 | 2,370 | 6,174 |
|  | V2 | 2,417 | ,999 | ,052 | -,019 | 4,853 |
| Based on estimated marginal estimates.  *. The difference in means is significant at the 0.05 level. | | | | | | |
| *. The difference in means is significant at the 0.05 level. | | | | | | |
| b. Adjustment for multiple comparisons: Bonferroni. | | | | | | |

Table 6

| **Pairwise comparison** | | | | | | |
| --- | --- | --- | --- | --- | --- | --- |
| Stratum corneum hydration SCH | | | | | | |
| (I) SCH | (J) SCH | Mean diff.(I-J) | SE | Sig.^b^ | 95% Confidence interval  for diff^b^ | |
|  |  |  |  |  | Lower limit | Upper limit |
| V1 | V2 | -1,820 | 1,236 | ,433 | -4,836 | 1,195 |
|  | V3 | -6,914 | 1,272 | <,001 | -10,017 | -3,811 |
| V2 | V1 | 1,820 | 1,236 | ,433 | -1,195 | 4,836 |
|  | V3 | -5,094 | 1,108 | <,001 | -7,796 | -2,391 |
| V3 | V1 | 6,914 | 1,272 | <,001 | 3,811 | 10,017 |
|  | V2 | 5,094 | 1,108 | <,001 | 2,391 | 7,796 |
| Based on estimated marginal estimates.  *. The difference in means is significant at the 0.05 level. | | | | | | |
| *. The difference in means is significant at the 0.05 level. | | | | | | |
| b. Adjustment for multiple comparisons: Bonferroni. | | | | | | |

Table 7

| **Pairwise comparison** | | | | | | |
| --- | --- | --- | --- | --- | --- | --- |
| pH-value | | | | | | |
| (I) pH | (J) pH | Mean diff.(I-J) | SE | Sig.^b^ | 95% Confidence interval  for diff^b^ | |
|  |  |  |  |  | Lower limit | Upper limit |
| V1 | V2 | ,175 | ,069 | ,041 | ,006 | ,344 |
|  | V3 | ,287 | ,058 | <,001 | ,146 | ,428 |
| V2 | V1 | -,175 | ,069 | ,041 | -,344 | -,006 |
|  | V3 | ,112 | ,061 | ,203 | -,036 | ,260 |
| V3 | V1 | -,287 | ,058 | <,001 | -,428 | -,146 |
|  | V2 | -,112 | ,061 | ,203 | -,260 | ,036 |
| Based on estimated marginal estimates.  *. The difference in means is significant at the 0.05 level. | | | | | | |
| *. The difference in means is significant at the 0.05 level. | | | | | | |
| b. Adjustment for multiple comparisons: Bonferroni. | | | | | | |

Table 8

| **Pairwise comparison** | | | | | | |
| --- | --- | --- | --- | --- | --- | --- |
| Epidermal thickness | | | | | | |
| (I) Epid thickn | (J) Epid thickn | Mean diff.(I-J) | SE | Sig.^b^ | 95% Confidence interval  for diff^b^ | |
|  |  |  |  |  | Lower limit | Upper limit |
| V1 | V2 | ,111 | 1,555 | 1,000 | -3,681 | 3,903 |
|  | V3 | 2,822 | 1,425 | ,152 | -,654 | 6,298 |
| V2 | V1 | -,111 | 1,555 | 1,000 | -3,903 | 3,681 |
|  | V3 | 2,711 | 1,706 | ,347 | -1,451 | 6,873 |
| V3 | V1 | -2,822 | 1,425 | ,152 | -6,298 | ,654 |
|  | V2 | -2,711 | 1,706 | ,347 | -6,873 | 1,451 |
| Based on estimated marginal estimates.  *. The difference in means is significant at the 0.05 level. | | | | | | |
| *. The difference in means is significant at the 0.05 level. | | | | | | |
| b. Adjustment for multiple comparisons: Bonferroni. | | | | | | |

Table 9

| **Pairwise comparison** | | | | | | |
| --- | --- | --- | --- | --- | --- | --- |
| Skin Roughness RA | | | | | | |
| (I) RA | (J) RA | Mean diff.(I-J) | SE | Sig.^b^ | 95% Confidence interval  for diff^b^ | |
|  |  |  |  |  | Lower limit | Upper limit |
| V1 | V2 | -,517 | ,374 | ,515 | -1,437 | ,402 |
|  | V3 | -1,435 | ,378 | <,001 | -2,363 | -,507 |
| V2 | V1 | ,517 | ,374 | ,515 | -,402 | 1,437 |
|  | V3 | -,918 | ,319 | ,016 | -1,702 | -,134 |
| V3 | V1 | 1,435 | ,378 | <,001 | ,507 | 2,363 |
|  | V2 | ,918 | ,319 | ,016 | ,134 | 1,702 |
| Based on estimated marginal estimates.  *. The difference in means is significant at the 0.05 level. | | | | | | |
| *. The difference in means is significant at the 0.05 level. | | | | | | |
| b. Adjustment for multiple comparisons: Bonferroni. | | | | | | |

Table 10

| **Pairwise comparison** | | | | | | |
| --- | --- | --- | --- | --- | --- | --- |
| Skin Roughness RZ | | | | | | |
| (I) RZ | (J) RZ | Mean diff.(I-J) | SE | Sig.^b^ | 95% Confidence interval  for diff^b^ | |
|  |  |  |  |  | Lower limit | Upper limit |
| V1 | V2 | ,107 | 1,528 | 1,000 | -3,646 | 3,860 |
|  | V3 | -4,866 | 1,535 | ,007 | -8,636 | -1,095 |
| V2 | V1 | -,107 | 1,528 | 1,000 | -3,860 | 3,646 |
|  | V3 | -4,973 | 1,310 | <,001 | -8,192 | -1,753 |
| V3 | V1 | 4,866 | 1,535 | ,007 | 1,095 | 8,636 |
|  | V2 | 4,973 | 1,310 | <,001 | 1,753 | 8,192 |
| Based on estimated marginal estimates.  *. The difference in means is significant at the 0.05 level. | | | | | | |
| *. The difference in means is significant at the 0.05 level. | | | | | | |
| b. Adjustment for multiple comparisons: Bonferroni. | | | | | | |

Table 11

| **Pairwise comparison** | | | | | | |
| --- | --- | --- | --- | --- | --- | --- |
| Skin stiffness | | | | | | |
| (I) stiff | (J) stiff | Mean diff.(I-J) | SE | Sig.^b^ | 95% Confidence interval  for diff^b^ | |
|  |  |  |  |  | Lower limit | Upper limit |
| V1 | V2 | -,001 | ,008 | 1,000 | -,020 | ,018 |
|  | V3 | ,025 | ,007 | ,002 | ,007 | ,042 |
| V2 | V1 | ,001 | ,008 | 1,000 | -,018 | ,020 |
|  | V3 | ,025 | ,008 | ,010 | ,005 | ,046 |
| V3 | V1 | -,025 | ,007 | ,002 | -,042 | -,007 |
|  | V2 | -,025 | ,008 | ,010 | -,046 | -,005 |
| Based on estimated marginal estimates.  *. The difference in means is significant at the 0.05 level. | | | | | | |
| *. The difference in means is significant at the 0.05 level. | | | | | | |
| b. Adjustment for multiple comparisons: Bonferroni. | | | | | | |

Table 12

| **Pairwise comparison** | | | | | | |
| --- | --- | --- | --- | --- | --- | --- |
| Skin elasticity | | | | | | |
| (I) elast | (J) elast | Mean diff.(I-J) | SE | Sig.^b^ | 95% Confidence interval  for diff^b^ | |
|  |  |  |  |  | Lower limit | Upper limit |
| V1 | V2 | ,040 | ,013 | ,007 | ,009 | ,071 |
|  | V3 | ,112 | ,013 | <,001 | ,079 | ,144 |
| V2 | V1 | -,040 | ,013 | ,007 | -,071 | -,009 |
|  | V3 | ,072 | ,013 | <,001 | ,040 | ,103 |
| V3 | V1 | -,112 | ,013 | <,001 | -,144 | -,079 |
|  | V2 | -,072 | ,013 | <,001 | -,103 | -,040 |
| Based on estimated marginal estimates.  *. The difference in means is significant at the 0.05 level. | | | | | | |
| *. The difference in means is significant at the 0.05 level. | | | | | | |
| b. Adjustment for multiple comparisons: Bonferroni. | | | | | | |

**Supplementary material for Table 2: Skin function and skin structure in women and infants at Visit 1, Visit 2, Visit3**

Table 13	Tests with paired groups for Infants

| **Tests with paired groups** | | | | | | | | | | | | | | | | | | | | | |
| --- | --- | --- | --- | --- | --- | --- | --- | --- | --- | --- | --- | --- | --- | --- | --- | --- | --- | --- | --- | --- | --- |
|  | | Paired Differences | | | | | | | | | | | T | | | df | | | Significance | | |
|  |  | mean | | SD | | SE mean | | 95% Confidence  interval of difference | | | |  | | |  | | | 1-sided p | | | 2-sided p |
|  |  |  |  |  |  |  |  | lower limit | | upper limit | |  | | |  | | |  | | |  |
| Pair 1 | EASI Total V2 - EASI Total_V3 | -.06147 | .67795 | | .06494 | | -.19018 | | .06725 | | -.947 | | | 108 | | | .173 | | | .346 | |
| Pair 2 | Skin elasticity_MEAN_Baby_V2 –  Skin elasticity_MEAN_B_V3 | -.06315 | .16795 | | .01751 | | -.09793 | | -.02837 | | -3.607 | | | 91 | | | <.001 | | | <.001 | |
| Pair 3 | Mean_epi_thickness_Baby_V2 - Mean_epi_thickness_Baby_V3 | -.27764 | 19.41934 | | 2.17115 | | -4.59920 | | 4.04392 | | -.128 | | | 79 | | | .449 | | | .899 | |
| Pair 4 | Mean_skin stiffness _Baby_V2 – Mean_skin stiffness _Baby_V3 | .02076 | .10521 | | .01097 | | -.00103 | | .04255 | | 1.893 | | | 91 | | | .031 | | | .062 | |
| Pair 5 | pH_mean_Baby_V2 - pH_mean_Baby_V3 | -.13226 | .55348 | | .05739 | | -.24625 | | -.01827 | | -2.304 | | | 92 | | | .012 | | | .023 | |
| Pair 6 | RA-mean_Baby_V2 –  RA-mean_Baby_V3 | .18468 | 3.30024 | | .38365 | | -.57992 | | .94929 | | .481 | | | 73 | | | .316 | | | .632 | |
| Pair 7 | RZ_mean_Baby_V2 - RZ_mean_Baby_V3 | 2.22523 | 13.15819 | | 1.52961 | | -.82328 | | 5.27373 | | 1.455 | | | 73 | | | .075 | | | .150 | |
| Pair 8 | SCH_mean_Baby_V2 - SCH_mean_Baby_V3 | -4.35532 | 15.63455 | | 1.61258 | | -7.55758 | | -1.15305 | | -2.701 | | | 93 | | | .004 | | | .008 | |
| Pair 9 | TEWL_mean_Baby_V2 - TEWL_mean_Baby_V3 | -.78830 | 9.10858 | | .93948 | | -2.65392 | | 1.07732 | | -.839 | | | 93 | | | .202 | | | .404 | |
