# Supplementary material for: Maternal and Infant Skin Microbiome Synchrony and Divergence: A Longitudinal Study of Skin Microbial Ecology and Early‐Life Assembly
Source: Exp Dermatol. 2026 Aug 21;35(8):e70349. doi: 10.1111/exd.70349 (PMC13498444; doi:10.1111/exd.70349)
Supplement: Supplementary file 1 [file EXD-35-e70349-s001.zip › Supplementary material_Table B.docx]

Supplementary material

Table 2 Correlation_Individual_external factors and Microbiom

| **Correlations** |  |  |  |  |  |  |  |  |  |  |  | |  |  |  |  |  |  |  |  |  |  |  |
| --- | --- | --- | --- | --- | --- | --- | --- | --- | --- | --- | --- | --- | --- | --- | --- | --- | --- | --- | --- | --- | --- | --- | --- |
|  | Alpha.B.V2 | Alpha.B.V3 | Alpha.M.V1 | Alpha.M.V2 | Alpha.M.V3 | Shannon.B.V2 | Shannon.B.V3 | Shannon.M.V1 | Shannon.M.V2 | Shannon.M.V3 | Taking of antibiotics | | Taking of antibiotics | Taking of antibiotics _M_V3 | Course of delivery_B_V2 | Vaginal seeding_B_V2 | Newborn nutrition_B_V2 | Newborn nutrition _B_V3 | dcn_pets_v3 | dcn_pets_v2 | dcn_caregiver_v2 | dcn_caregiver_v3 | Gestational age at birth_B_V2 |
| Alpha.B.V2 | 1 |  |  |  |  |  |  |  |  |  |  | |  |  |  |  |  |  |  |  |  |  |  |
| Alpha.B.V3 | 0,032 | 1 |  |  |  |  |  |  |  |  |  | |  |  |  |  |  |  |  |  |  |  |  |
| Alpha.M.V1 | 0,189 | -0,042 | 1 |  |  |  |  |  |  |  |  | |  |  |  |  |  |  |  |  |  |  |  |
| Alpha.M.V2 | ,639^**^ | -0,098 | 0,159 | 1 |  |  |  |  |  |  |  | |  |  |  |  |  |  |  |  |  |  |  |
| Alpha.M.V3 | 0,044 | ,455^**^ | 0,151 | 0,05 | 1 |  |  |  |  |  |  | |  |  |  |  |  |  |  |  |  |  |  |
| Shannon.B.V2 | ,447^**^ | -0,02 | 0,092 | ,296^**^ | -0,144 | 1 |  |  |  |  |  | |  |  |  |  |  |  |  |  |  |  |  |
| Shannon.B.V3 | 0,13 | ,513^**^ | 0,012 | 0,036 | 0,032 | 0,106 | 1 |  |  |  |  | |  |  |  |  |  |  |  |  |  |  |  |
| Shannon.M.V1 | 0,062 | 0,023 | ,516^**^ | 0,118 | 0,075 | 0,114 | -0,052 | 1 |  |  |  | |  |  |  |  |  |  |  |  |  |  |  |
| Shannon.M.V2 | ,283^**^ | -0,122 | 0,08 | ,419^**^ | -0,075 | ,375^**^ | -0,025 | ,296^**^ | 1 |  |  | |  |  |  |  |  |  |  |  |  |  |  |
| Shannon.M.V3 | 0,114 | ,240^*^ | -0,008 | 0,127 | ,536^**^ | -0,03 | 0,083 | -0,032 | 0,08 | 1 |  | |  |  |  |  |  |  |  |  |  |  |  |
| Taking of antibiotics_M_V1 | -0,092 | -0,018 | -0,053 | -0,098 | 0,021 | 0,058 | -0,172 | -0,013 | 0,14 | 0,11 | 1 | |  |  |  |  |  |  |  |  |  |  |  |
| Taking of antibiotics_M_V2 | 0,059 | 0,033 | -0,013 | 0,123 | -0,027 | 0,177 | -0,101 | -0,169 | -0,156 | -0,181 | 0,093 | | 1 |  |  |  |  |  |  |  |  |  |  |
| Taking of antibiotics _M_V3 | -0,071 | 0,019 | -0,091 | 0,091 | 0,197 | -,216^*^ | -,214^*^ | 0,045 | -0,035 | 0,124 | -0,037 | | -0,076 | 1 |  |  |  |  |  |  |  |  |  |
| Course of delivery_B_V2 | 0,097 | 0,126 | 0,002 | 0,109 | -0,083 | 0,13 | -0,04 | -0,013 | -0,115 | -0,204 | 0,028 | | ,426^**^ | -0,111 | 1 |  |  |  |  |  |  |  |  |
| Vaginal seeding_B_V2 | -0,034 | 0,161 | -0,198 | -0,232 | -0,096 | -0,291 | 0,116 | -0,291 | -,468^*^ | 0,191 | -0,132 | | 0,022 | .^c^ | .^c^ | 1 |  |  |  |  |  |  |  |
| Newborn nutrition_B_V2 | -0,029 | ,207^*^ | 0,011 | 0,018 | 0,021 | 0,076 | 0,182 | -0,011 | 0,059 | -,245^*^ | -0,074 | | 0,007 | 0,097 | ,218^*^ | -0,05 | 1 |  |  |  |  |  |  |
| Newborn nutrition _B_V3 | -0,186 | 0,087 | -,248^*^ | -0,198 | -0,066 | 0,049 | 0,049 | -0,032 | 0,03 | -0,042 | 0,039 | | 0,007 | -0,136 | 0,011 | -0,17 | 0,082 | 1 |  |  |  |  |  |
| dcn_pets_v3 | -0,042 | -0,02 | 0,091 | 0,025 | 0,046 | -0,12 | 0,109 | 0,148 | -0,046 | 0,051 | 0,028 | | 0 | -0,021 | 0 | .^c^ | ,209^*^ | -0,119 | 1 |  |  |  |  |
| dcn_pets_v2 | -0,079 | -0,052 | 0,055 | -0,047 | -0,086 | -0,152 | 0,061 | 0,048 | -0,1 | -0,068 | 0,018 | | 0 | -,283^**^ | 0 | .^c^ | 0 | 0,19 | ,355^**^ | 1 |  |  |  |
| dcn_caregiver_v2 | -0,15 | -0,099 | 0,004 | -0,023 | -0,139 | -0,14 | -,206^*^ | 0,027 | -0,093 | -0,025 | 0,193 | | -0,004 | 0,077 | -0,12 | -0,055 | -0,177 | -0,014 | -0,147 | 0 | 1 |  |  |
| dcn_caregiver_v3 | -0,016 | -0,102 | 0,053 | 0,021 | -,203^*^ | -0,006 | -0,14 | 0,018 | -0,055 | -,218^*^ | -0,045 | | -0,072 | 0,042 | -0,083 | 0,411 | -0,004 | 0,001 | -0,027 | 0,016 | ,382^**^ | 1 |  |
| Gestational age at birth_B_V2 | 0,03 | 0,029 | -0,07 | 0,015 | 0,065 | -0,169 | -0,078 | -0,069 | -,213^*^ | 0,114 | -,288^**^ | | 0,061 | 0,113 | -0,05 | -0,012 | -0,017 | -0,099 | ,208^*^ | ,297^**^ | -0,052 | -0,061 | 1 |
| **. The correlation is significant at the 0.01 level (two-tailed). | | | | | | | | | | | |  |  |  |  |  |  |  |  |  |  |  |  |
| *. The correlation is significant at the 0.05 level (two-tailed). | | | | | | | | | | | |  |  |  |  |  |  |  |  |  |  |  |  |
| c. Cannot be calculated, as at least one of the variables is constant. | | | | | | | | | | | |  |  |  |  |  |  |  |  |  |  |  |  |
