# Supplementary material for: Maternal and Infant Skin Microbiome Synchrony and Divergence: A Longitudinal Study of Skin Microbial Ecology and Early‐Life Assembly
Source: Exp Dermatol. 2026 Aug 21;35(8):e70349. doi: 10.1111/exd.70349 (PMC13498444; doi:10.1111/exd.70349)
Supplement: Supplementary file 1 [file EXD-35-e70349-s001.zip › Supplementary material_Table N.docx]

**Supplementary material**

**Table 14 Negative controls Top5 Taxa**

| **Genus** | **mean_read_count_**  **detected_controls** | **mean_read_count_**  **all_controls** | **controls_detected** | **controls_**  **with_zero_reads** |
| --- | --- | --- | --- | --- |
| Staphylococcus | 215 | 167 | 14 | 4 |
| Pelomonas | 270 | 165 | 11 | 7 |
| Corynebacterium | 200 | 145 | 13 | 5 |
| Lactobacillus | 379 | 63 | 3 | 15 |
| Aquabacterium | 210 | 58 | 5 | 13 |
| All remaining genera/OTUs, including unclassified | 483 | 483 | 18 | 0 |
